# Selenium Nanoparticles: Synthesis, Stability and In Vitro Evaluation in Human Lens Epithelial Cells

**DOI:** 10.3390/pharmaceutics17091157

**Published:** 2025-09-03

**Authors:** Lulwah Al-Bassam, Mohammed M. Naiyer, Christopher J. Morris, Steve Brocchini, Gareth R. Williams

**Affiliations:** UCL School of Pharmacy, University College London, 29-39 Brunswick Square, London WC1N 1AX, UK; lulwah.albassam.20@ucl.ac.uk (L.A.-B.); m.naiyer@ucl.ac.uk (M.M.N.); chris.morris@ucl.ac.uk (C.J.M.); s.brocchini@ucl.ac.uk (S.B.)

**Keywords:** nanoparticle drug delivery, d-α-tocopheryl polyethylene glycol succinate, ocular selenium, antioxidant therapy

## Abstract

**Background/Objectives**: Oxidative stress plays a critical role in the development of ocular diseases such as cataracts. Selenium nanoparticles (SeNPs) offer antioxidant benefits with low toxicity. This study aimed to evaluate the antioxidant activity of SeNPs coated with D-α-tocopheryl polyethylene glycol succinate (TPGS) in human lens epithelial (HLE) cells. **Methods**: SeNPs were synthesised by reducing sodium selenite with ascorbic acid in the presence of TPGS. Physicochemical characterisation was carried out using dynamic light scattering to assess size and surface charge. Antioxidant activity was measured by a 2,2-diphenyl-1-picrylhydrazyl (DPPH) assay. Cytocompatibility was assessed on adult retinal pigment epithelial (ARPE-19) and HLE cells using PrestoBlue. Functional antioxidant performance was determined through enzymatic assays for glutathione peroxidase (GPx), thioredoxin reductase (TrxR), and glutathione (GSH), and lipid peroxidation was assessed using malondialdehyde (MDA) quantification. Catalase mimicry was evaluated under 3-amino-1,2,4-triazole (3-AT)-induced inhibition. **Results**: The optimal SeNP formulation had an average hydrodynamic diameter of 44 ± 3 nm, low PDI (<0.1), and a surface charge of −15 ± 3 mV. These TPGS-SeNPs demonstrated strong radical scavenging (EC_50_ ≈ 1.55 µg/mL) and were well tolerated by ARPE-19 cells (IC_50_ = 524 µg/mL), whereas HLE cells had a narrower biocompatibility window (≤0.4 µg/mL, IC_50_ = 2.2 µg/mL). Under oxidative stress, SeNPs significantly enhanced GPx and TrxR activity but did not affect GSH or MDA levels. No catalase-mimetic activity was observed. **Conclusions**: TPGS-SeNPs exhibit potent antioxidant enzyme modulation under stress conditions in HLE cells. Although not affecting all oxidative markers, these nanoparticles show promise for non-invasive strategies targeting lens-associated oxidative damage, including cataract prevention.

## 1. Introduction

Oxidative stress is a critical factor in the progression of chronic ocular disorders such as cataracts, diabetic retinopathy, and age-related macular degeneration [1]. The lens, in particular, is highly susceptible to oxidative damage due to its unique characteristics: long-lived proteins with minimal turnover, constant exposure to light, and decreasing antioxidant capacity with age leading to cataracts [2]. The accumulation of reactive oxygen species (ROS) leads to protein oxidation, aggregation, and ultimately, lens opacity [3,4]. This pathological process accelerates with advancing age as antioxidant levels decline, resulting in progressive damage to ocular structures [3].

There are no approved drugs that prevent oxidative damage to the ocular lens, and lifestyle advice remains the mainstay of promoting ocular health. Among potential therapeutic agents for counteracting oxidative stress, selenium has emerged as a promising candidate due to its incorporation in numerous antioxidant defence systems [5]. Selenium is a fundamental element to more than 25 selenoproteins, including glutathione peroxidase (GPx), selenoprotein P, and thioredoxin reductase (TrxR), which facilitate antioxidant defence, immunological regulation, and numerous metabolic activities [6,7,8,9]. GPx, a vital cytosolic enzyme in the eye, mitigates oxidative stress, prevents apoptosis, and enhances cell survival [10,11,12,13]. Increased GPx levels improve lens cell survival under stress [14] and prevent DNA damage by reducing ROS levels [15,16]. Studies have demonstrated the significance of GPx activity in, for instance, the progression of cataracts, particularly nuclear cataracts [17,18,19], with regular selenium consumption potentially slowing cataract development [20].

Similarly, TrxR is important in keeping the lens in a reduced state [21] and balances cellular redox reactions [22,23]. This enzyme is present in both lens epithelium and fibre cells [24], but decreases in cataractous lenses [24,25]. Additional antioxidant enzymes, such as catalase, also play protective roles in the lens epithelium [26], though their activity diminishes with age. Meanwhile, lipid peroxidation byproducts like malondialdehyde (MDA) accumulate with age and may contribute to cataractogenesis through protein cross-linking mechanisms [27,28]. Strategies to counteract this process focus primarily on preventing lipid peroxidation through dietary antioxidants like vitamins C and E and lutein, which neutralise free radicals before they can initiate membrane damage [29]. Maintaining adequate selenoprotein levels and supporting endogenous antioxidant enzyme systems can also help prevent the accumulation of these harmful byproducts and preserve lens transparency [2].

While oral selenium supplementation can enhance the systemic expression and functioning of selenoproteins and enzymes [30,31,32,33], the development of topical ocular selenium formulations represents a particularly valuable research direction for ocular conditions like cataracts. Only a limited number of studies have investigated the impact of topically applied selenium on ocular health [34,35,36,37,38]. Localised delivery could maximise therapeutic benefit while minimising systemic exposure. However, challenges remain in delivering selenium effectively to ocular tissues due to physiological barriers and the need for stable, bioavailable formulations.

Nanoparticle-based systems offer many advantages for ocular drug delivery, including enhanced epithelial permeation, controlled release, and improved bioavailability [39,40]. Selenium nanoparticles (SeNPs) can be produced through various methods including biological, chemical, and green synthesis procedures [41,42,43]. The most common approach involves chemical reduction of selenium salts into elemental Se, using agents like ascorbic acid, with stabilising agents controlling particle size and preventing aggregation [42]. These synthesis methods allow for customisation of nanoparticle properties to optimise ocular delivery and therapeutic efficacy.

For ocular applications, maintaining nanoparticle colloidal stability is essential to ensure consistent dosing [44]. D-α-tocopherol polyethylene glycol succinate (TPGS), a water-soluble vitamin E derivative, can be used to this end [45]. This nonionic surfactant demonstrates potential to improve drug absorption through biological barriers, including the ocular barrier [46,47,48]. TPGS-based micelles have shown enhanced drug permeation across and retention inside the porcine conjunctival epithelium [49] and cornea [50], with favourable tolerance in ocular tissue [50,51,52,53,54]. However, TPGS-stabilised SeNPs have not been widely explored in the literature.

Based on these insights, we hypothesised that SeNPs surface-functionalised with TPGS would be able to protect against oxidative stress. This study aims to explore the potential of using SeNPs for ocular conditions, evaluating their biocompatibility and ability to enhance antioxidant enzyme activities. Our approach represents a potential non-invasive strategy to slow the progress of cataractogenesis.

## 2. Materials and Methods

### 2.1. Materials

Sodium selenite (CAS 10102-18-8), ascorbic acid (AA; CAS 50-81-7), and α-tocopherol succinate (TPGS; CAS 9002-96-4) were obtained from Sigma Aldrich, Gillingham, UK. Solvents comprised ultrapure water (UPW, Purite, Thame, UK) and 99.8% ethanol (absolute analytical; Thermo Fisher, Loughborough, UK). Human retinal pigment epithelial cells (ARPE-19) and Eagle’s minimum essential medium (EMEM) were sourced from ATCC (Manassas, VA, USA). Dulbecco’s Modified Eagle’s Medium-F12 (DMEM), Dulbecco’s phosphate-buffered saline (DPBS), foetal bovine serum, penicillin–streptomycin solution, Accutase (0.025% trypsin and 0.01% EDTA), Live/dead™ cell imaging kits (488/570), a Pierce™ BCA protein assay kit, and Pierce protease and phosphatase inhibitor mini tablets were purchased from Thermo Fisher (UK). Selenomethionine was obtained from MedChem Express (Monmouth Junction, NJ, USA). Glutathione peroxidase assay kits were procured from Cayman chemical (Ann Arbor, MI, USA). PrestoBlue and phenylmethylsulfonyl fluoride (PMSF) were purchased from Thermo Fisher (UK). NP-40 lysis buffer was acquired from Alfa Aesar (Heysham, UK). SV40 human lens epithelial cells were purchased from Addexbio at passage 15. An Amplite^®^ fluorimetric malondialdehyde (MDA) quantitation kit was obtained from AAT Bioquest, Pleasanton, CA, USA. Thioredoxin reductase and GSH/GSSG assay kits were sourced from Abcam, Cambridge, UK. Trichloroacetic acid (TCA) was purchased from Avantor (Radnor, PA, USA). Polystyrene solid black 96-well plates were sourced from Corning (Corning, NY, USA). White polystyrene flat-bottomed 96-well plates (Nunclon Delta-Treated) and black polystyrene 96-well plates with clear bottoms were obtained from Thermo Fisher, UK. Both 2,2-diphenyl-1-picrylhydrazyl and 3-amino-1,2,4-triazole were obtained from Sigma-Aldrich, UK.

### 2.2. Methods

TPGS-SeNPs were prepared by reduction of sodium selenite using AA in the presence of TPGS. Briefly, 5 mM sodium selenite was dissolved in distilled water (5 mL) in a 14 mL glass vial. In a separate 14 mL glass vial, 5 mL of an aqueous AA solution (65 mM) was freshly prepared and covered with aluminium foil to prevent light-mediated oxidation. In a third glass vial, TPGS (0.33 mM) was dissolved in 5 mL water or ethanol (40 °C) under magnetic stirring.

The influence of reagent addition order on SeNP size and uniformity was examined. In Process A, an aqueous TPGS solution was added to the sodium selenite solution and stirred (900 rpm) for 10 min before the AA was added dropwise over ~2 min. The mixture was placed in a bath sonicator for 10 min (Kerry PUL55, Hitchin, UK). In Process B, TPGS was dissolved in ethanol and mixed with aqueous sodium selenite for 10 min, then aqueous AA was added dropwise. The mixture was next placed in a rotary evaporator to remove the ethanol (3 min), and the colloidal solution was placed in a bath sonicator for 10 min. Process C used the same solutions as process B, but the ethanolic TPGS solution was initially subjected to rotary evaporation to remove the ethanol and give a thin film. This was followed by rehydration with aqueous sodium selenite (5 mL, 5 mM). Finally, AA solution was added dropwise with stirring. Uncoated SeNPs were also prepared, by adding AA (5.0 mL, 65 mM in water) dropwise to a 5 mL solution of sodium selenite (5 mM in water) under the same magnetic stirring setting.

Following sonication, colloidal solutions were transferred to 50 mL polycarbonate centrifuge tubes. The suspensions were centrifuged twice in deionised water for 30 min, at 18,000 rpm and 4 °C. After the first round, the supernatant was removed and replaced with deionised water. The final solution was reconstituted with 14 mL water and filtered twice through 0.22 µm syringe filters. Some samples were subsequently freeze-dried with the aid of a SP VirTis Advantage Benchtop Freeze Dryer (SP Scientific, Warminster, PA, USA). The sample was initially frozen at −60 °C for 48 h, followed by sublimation under vacuum to remove the ice. Once most of the ice had sublimed during the primary drying phase, the temperature was increased to 20 °C for 2 h to remove most of the remaining bound water.

#### 2.2.1. Dynamic Light Scattering (DLS) and Stability Measurements

The size distribution and zeta potential analyses were performed using a Zetasizer Ultra instrument and Zx explorer software (Malvern Panalytical, version 2.0.1.1, Malvern, UK). A 1 mL aliquot of each sample of TPGS-SeNPs was diluted with 1 mL of deionised water; then, 0.7 mL was placed in a disposable polycarbonate folded capillary zeta cell (Malvern Instruments, DTS1070, UK), and loaded into the instrument. These cells allow the determination of both size and zeta potential, and were used for both measurements. The mean intensity-weighted diameter of the individual particle populations and mean zeta potential values were recorded as mean ± SD from three batches of NPs. Formulations were stored with refrigeration (5 ± 3 °C) and at room temperature (25 ± 2 °C) and measurements recorded periodically to assess stability.

#### 2.2.2. Transmission Electron Microscopy (TEM)

An aliquot of SeNP suspension was added to a copper grid coated with a carbon membrane (TAAB, Aldermaston, UK; F196/100) and allowed to dry. Images were recorded on a CM120 Bio Twin TEM (Philips/FEI, Eindhoven, The Netherlands).

#### 2.2.3. Fourier Transform Infrared (FTIR) Spectroscopy

Freeze-dried samples were analysed on a Spectrum 100 spectrometer (Perkin Elmer, Waltham, MA, USA). Spectra were collected over the range of 650–4000 cm^−1^ at a resolution of 4 cm^−1^. Fifty scans were collected in attenuated total reflectance mode, using a few mg of powdered sample.

#### 2.2.4. X-Ray Diffraction (XRD)

XRD was conducted using a Miniflex 600 diffractometer (Rigaku, Tokyo, Japan) supplied with Cu Kα radiation (λ = 1.5418 nm). Patterns were gathered over the 2θ range 3–60°, at 40 kV, 15 mA, and 0.5 °/min. Powdered samples of a few mg were loaded into flat sample holders and data collected in Bragg–Brentano geometry.

#### 2.2.5. Inductively Coupled Plasma-Optical Emission Spectroscopy (ICP-OES)

A 720-ES ICP-OES instrument with an autosampler (Agilent Technologies, formerly Varian, Cheadle, UK) was used for these measurements. The supernatants from SeNP synthesis were centrifuged twice and filtered using a 0.22 µm filter before analysis.

#### 2.2.6. 2,2-Diphenyl-1-Picrylhydrazyl (DPPH) Assay

The radical scavenging ability of TPGS-SeNPs against DPPH was measured as described by Cheng [55], with slight modifications. In a 96-well plate, 100 µL of each SeNP sample was mixed with 100 µL of methanol, then serially diluted with methanol to ensure a total volume of 100 µL per well. We added 50 µL of DPPH solution (0.1 mM) to all wells. A mixture of 100 μL of methanol with 50 µL of DPPH was used as a negative control. AA and methanol (100 µL) acted as a positive control and blank, respectively. The plate was incubated in the dark for 30 min. The absorbance was then measured at 517 nm in a Spectramax M2e microplate reader (Molecular Devices, Wokingham, UK). The radical scavenging activity (*RSC*%) was calculated using the following formula:

(1) $$RSC\%=\;\left[\left(A_{c}-A_{t}\right)/A_{c}\right]\;\times 100\;\;$$

where A_c_ is the absorbance of the control and A_t_ is the absorbance of the test sample [56,57,58].

#### 2.2.7. Cell Viability

The cytotoxicity of the SeNPs was evaluated using human retinal pigment epithelial (ARPE-19) and SV40 human lens epithelial (HLE) cell lines. ARPE-19 cells were cultured in a 75 cm^2^ tissue culture flask at 37 °C under a 5% CO_2_ humidified atmosphere in DMEM-F12 containing 5% *v*/*v* penicillin–streptomycin solution and 10% *v*/*v* foetal bovine serum (FBS). The cells were passaged at 70–80% confluency. HLE cells were cultured in EMEM containing 5% *v*/*v* penicillin–streptomycin solution and 20% *v*/*v* FBS. Culturing was performed in a 75 cm^2^ tissue culture flask at 37 °C under a 5% CO_2_ humidified atmosphere (18, 20–22 passages).

For assays, cells were loaded at 1 × 10^4^ cells/well (100 µL/well) in black polystyrene 96-well plates with clear bottoms. The cells were allowed to adhere for 24 h in a 5% CO_2_ incubator at 37 °C. The medium was then removed and replaced with fresh media containing different concentrations of SeNPs. The cells were subjected to a further 24 h incubation period. Then, the cell viability was evaluated using a PrestoBlue test kit following the manufacturer’s directions. A SpectraMax M2e microplate reader (Molecular Devices, Wokingham, UK) was set to read the fluorescence at 560–590 nm from the bottom of the plates [59]. Three independent experiments with 6 replicate wells in each were performed, and cell viability was calculated using Equation (2). Results are presented as mean ± standard deviation.

(2) $$Cell\;viability\;\%=\frac{\left(Fluorescence\;of\;treated\;cells-fluorescence\;of\;background\right)}{\left(Untreated\;cells\;controlfluorescence-background\;fluorescence\right)}x100$$

#### 2.2.8. ATP Assay

The luminescent ATP detection assay kit (ab113849) was used for quantifying the concentration of ATP present in the cells. HLE cells were cultured at 1 × 10^4^ cells/well (100 µL/well) in white polystyrene, flat-bottomed, 96-well plates for 24 h. The medium was aspirated and replaced with 100 μL of fresh media supplemented with SeNPs (0.1–8 μg/mL), and the cells were incubated for a further 24 h. The ATP assay was then performed in accordance with the instructions provided by the manufacturer. The kit is for one plate (*n* = 4 replicate wells). Luminescence was quantified using the SpectraMax M2e plate reader. The ATP concentrations in the test samples were determined from standard curves and normalised to the protein content assessed using a Pierce BCA Protein Assay Kit.

#### 2.2.9. BCA Assay

The BCA test was conducted using 96-well plates in accordance with the manufacturer’s instructions. The reagent was prepared in accordance with the instructions supplied by Pierce. Bovine serum albumin (BSA) standards were prepared in NP-40 buffer (2 mg/mL) in 1.5 mL Eppendorfs.

#### 2.2.10. Live/Dead Assay

HLE cells were seeded into 96-well tissue culture plates at a density of 1 × 10^4^ cells (100 μL) per well. Following a 24 h incubation, the medium was removed and replaced with 100 µL of EMEM supplemented with SeNPs at 0.05, 0.1, and 0.2 μg/mL along with hydrogen peroxide (300 µM), and incubated for a further 24 h. Ethanol was used as a positive control. After the second incubation period, the medium was aspirated, and the cells were incubated with a combination of serum-free medium and dye (80 µL). The latter was prepared using a Live/DEAD™ cell imaging kit (488/570 nm), by adding 1 mL of calcein acetoxymethyl, a live cell indicator, and 1 µL of dimeric cyanine nucleic acid stain BOBO-3 iodide, a dead cell indicator, to an equivalent quantity of serum-free medium. Following a short incubation period of 30 min at 20 °C, the cells were imaged with an EVOS XL microscope (Thermo Fisher, Waltham, MA, USA).

#### 2.2.11. GPx Assay

Each sample consisted of 2 × 10^6^ HLE cells seeded in a T75 flask in 12 mL complete EMEM. The flasks were incubated for 24 h, and then the medium was aspirated and replaced with 12 mL of fresh media containing different concentrations of SeNPs (0.05–0.2 µg/mL). This was then followed with another 24 h incubation. Next, the medium was removed and replaced with 10 mL of serum-free medium augmented with H_2_O_2_ (600 µM) for 4 h. Next, the cells were washed with DPBS and collected using 3 mL of accutase. The cells were centrifuged to create a pellet, which was rinsed with 1 mL of cold DPBS and centrifuged at 12,000 rpm (Micro Star 17 microcentrifuge, VWR, Radnor, PA, USA). Each pellet was lysed with 120 µL of lysis buffer and transferred to a PCR tube (Eppendorf, Stevenage, UK). The lysis buffer components were varied from those initially suggested by the manufacturer and comprised NP-40 lysing buffer, PI cocktail, Pierce protease and phosphatase inhibitor mini tablet solution, and Triton X100 (79.5% *v*/*v* NP-40 lysing buffer, 10% *v*/*v* PI cocktail, 10% *v/v* Pierce protease and phosphatase inhibitor mini tablet solution, and 0.5% *v*/*v* Triton X100). Cells were incubated on ice with periodic vortexing and were further lysed by repeated passage through an XG insulin needle. The lysates were incubated for 45 min on ice. The Eppendorf tubes were then spun at 10,000× *g* for 15 min, and the supernatants were transferred into fresh tubes before being frozen at −80° C for later GPx and BCA assays.

GPx levels were determined using a GPx assay kit (Cayman) following the supplier’s instructions. Absorbance was read at 340 nm using a SpectraMax M2e plate reader (Molecular Devices, Wokingham, UK), with measurements taken every minute for 15 min. The protein concentration was quantified via the BCA assay and used to normalise the data.

#### 2.2.12. GSH Assay

The effect of SeNPs on GSH levels was determined using a GSH detection kit following the supplier’s instructions. Each sample consisted of 2 × 10^6^ HLE cells seeded in a T75 flask containing 12 mL of complete EMEM. Cell culture and recovery was performed using the same protocols as for the GPx assay. A lysis buffer (200 µL/sample) was prepared using PMSF, protease inhibitors, and Triton X according to the manufacturer’s instructions. After lysis, the supernatant was frozen before further analysis. Fluorescence was monitored at Ex/Em = 490/520 nm with a SpectraMax M2e plate reader.

#### 2.2.13. MDA Assay

The Amplite^®^ fluorimetric quantitation kit was used to measure MDA levels in the supernatant of cells treated with SeNPs. Cell culture was performed as for the GPx assay. Analysis was performed using polystyrene, solid black, 96-well plates (Corning). We prepared 50 µL of MDA solution and added that to the standard wells in duplicate, and 50 µL of the analyte was added to the sample wells, also in duplicate. The assay was carried according to the manufacturer’s instructions, and the fluorescence was measured at an excitation wavelength of 365 nm and an emission wavelength of 435 nm at two time points (20 and 60 min).

#### 2.2.14. TrxR Assay

HLE samples were prepared in a similar way to those for GPx assays except that at the end of the experiment, the samples were homogenised in 200 μL of cold thioredoxin reductase assay buffer containing a Pierce protease and phosphatase inhibitor solution (1 mini tablet in 10 mL ultrapure water). The homogenisation was completed by needle shearing on ice. Post-homogenisation, the samples were centrifuged at 10,000× *g* for 15 min at 4 °C, and the supernatant was then kept on ice. From this supernatant, two sets of samples were tested with or without TrxR inhibitor. For the former, 10 μL of thioredoxin reductase inhibitor was added to one sample set to assess background enzyme activity, while 10 μL of TrxR assay buffer was added to the latter sample set. In both cases, this was followed by thorough mixing. We added 40 μL of a reaction mixture supplied with the kit, prepared according to the manufacturer’s instructions, to each test sample and positive control, again followed by thorough mixing. The total protein concentration was quantified via the BCA assay. Absorbance was measured at 412 nm at T_0_ to determine A_1t_ (samples without inhibitor) and A_1I_ (samples with TrxR inhibitor), then was measured again at T_20_ after incubating the reaction at 25 °C for 20 min, to derive A_2t_ and A_2I_.

ΔA_412 nm_ = (A_2t_ − A_2I_) − (A_1t_ − A_1t_) (3)

(4) $$TrxR\;activity=\frac{∆B}{(T_{20}-T_{0})\times V}\;\times \;sample\;dilution\;factor=\frac{\frac{nmol}{min}}{mL}=mU/mL$$

ΔA_214nm_ is applied to the 5-thio-2-nitrobenzoic acid (TNB) standard curve (in nmol) and is used to obtain ∆B (nmol of TNB). T_0_ is the time of the first reading (A_1t_ and A_1I_). T_20_ is the time of the second reading (A_2t_ and A_2I_). V is the pretreated sample volume added into the reaction well.

#### 2.2.15. 3-AT Assay

To determine if SeNPs protect the HLE cell line from oxidative stress by catalase-mimetic activity, the catalase inhibitor 3-amino-1,2,4-triazole (3-AT, Sigma Aldrich, UK) was used, as documented in the literature with modifications [60,61]. HLE cells were seeded in complete EMEM in 96-well plates at a density of 1 × 10^4^ cells/well (100 µL/well) and left for 24 h. The medium was aspirated and then the cells were incubated with 100 μL of medium containing SeNPs at a concentration of 0.05–0.2 μg/mL. A negative control (no treatment) and a positive control (3-AT alone) were implemented. 24 h later, the medium was aspirated, and the cells were washed once with 100 μL DPBS before being incubated with 100 μL of 3-AT (100 mM) for 24 h. Following the catalase inhibitor treatment, the medium was removed, the cells were rinsed once with DPBS, and cell viability was assessed using the PrestoBlue assay as previously described. Cell viability was represented as a percentage of the negative control. The experiment was conducted four times with 5 replicates per plate (*N* = 4, *n* = 5).

#### 2.2.16. Statistical Analysis

Each experiment was repeated a minimum of three times. Statistical analysis was conducted using the GraphPad Prism 9 software (version 9) and applying analysis of variance (ANOVA) and a post hoc Tukey test. Statistical significance was determined at a threshold of * *p* < 0.05, ** *p* < 0.01, *** *p* < 0.001, **** *p* < 0.0001.

## 3. Results

### 3.1. NP Synthesis

**Figure 1:**
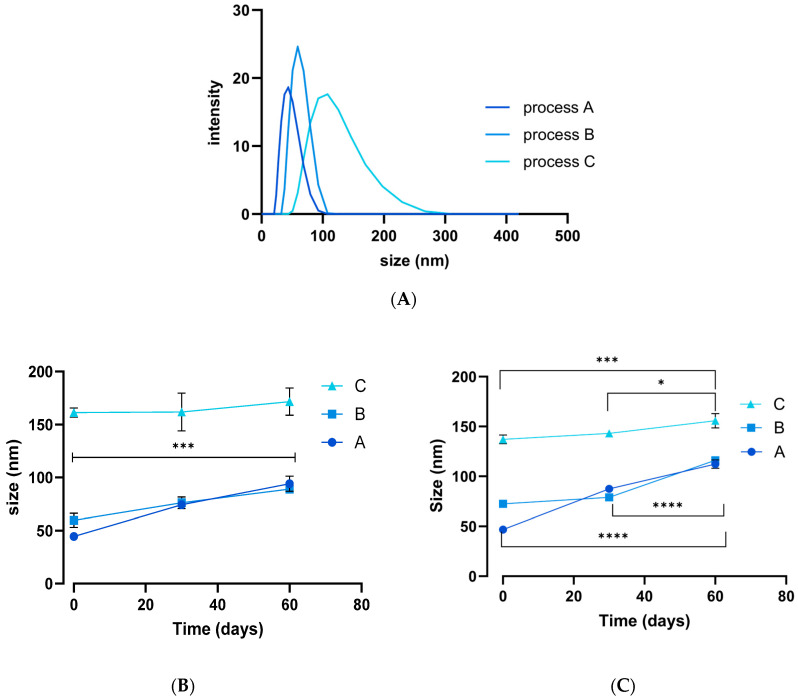
DLS data showing (**A**) the average particle size for each process immediately after synthesis, together with the change in size upon storage (**B**) in the fridge and (**C**) at room temperature. Data shown as mean ± S.D. (*n* = 3). * *p* < 0.05, *** *p* < 0.001, **** *p* < 0.0001. One-way ANOVA.

The mean size obtained of SeNPs prepared without TPGS was 371 ± 214 nm (PDI 0.3; Supplementary Information, Figure S1A). Following 7 days of storage at 2–8 °C, the mean size was 1330 ± 148 nm and the particles were highly polydisperse (PDI 0.7; Figure S1B), in agreement with previously published results [62]. Uncoated SeNPs clearly aggregate with time and hence are not suitable for use in biomedical applications. TPGS was thus introduced as a stabilising agent and three different synthesis approaches explored to generate NPs using varying water–ethanol mixtures. Figure 1A shows the DLS data obtained. The hydrodynamic diameter of process A (water-based) TPGS-SeNPs was initially 44 ± 3 nm but increased significantly over a 60-day storage period (Figure 1B,C). Similar trends were observed for process B (ethanol-based; initially 60 ± 1 nm) and process C (thin-film rehydration; initially 161 ± 3 nm) TPGS-SeNPs (Figure 1B). Over time, the solutions became turbid due to particle growth, but they remained colloidal and could be re-dispersed with gentle shaking. The observed PDI values were less than 0.1. The mean zeta potential obtained for all three process was in the range of −15 ± 3 to −27 ± 1 mV at day 0, −25 ± 5 to −38 ± 7 mV at day 30, and −20 ± 5 to −35 ± 3 mV at day 60.

**Figure 2:**
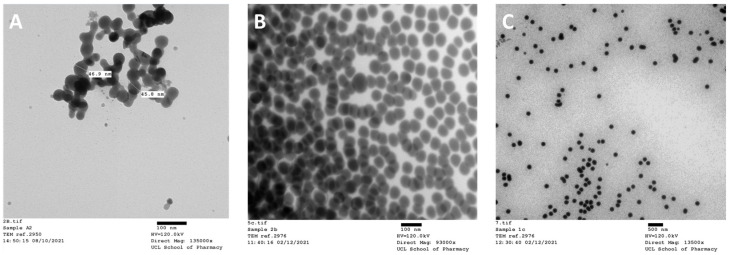
TEM images of the different TPGS-SeNPs for processes (**A**–**C**). Scale bar 100 nm for (**A**) and (**B**), 500 nm for (**C**).

TEM confirmed that the TPGS-SeNPs fabricated by the three processes were predominantly spherical in shape (Figure 2). The particles exhibited a uniform morphology with smooth surfaces. No differences in shape or surface features were observed among the samples fabricated by the three different processes. For the process A SeNPs, we find a size of 31 ± 8 nm by TEM, while process B gives 49 ± 5 nm and process C 130 ± 16 nm. The sizes observed by TEM are somewhat lower than those obtained by DLS, because of the particles being hydrated in the latter measurement. In addition, two populations of TPGS-SeNPs were observed in process C, with a large number of markedly smaller particles at 24 ± 4 nm (see Figure S2).

ICP-OES was used to quantify the amount of selenium in the supernatant (i.e., the amount of Se not incorporated into NPs) and determine the process yields. The NP yields based on Se content were found to be 62 ± 1%, 82 ± 1%, and 47 ± 1%, for processes A–C, respectively. XRD patterns showed the NPs from processes A and B to be amorphous (Figure S3), whereas crystalline material was seen with process C (Figure S4). The Bragg reflections observed did not match with any of the allotropes of Se (Figure S4), and instead the crystalline material was identified to be residual AA from the synthesis process (Figure S5). Given these observations and the changes in NP size, we selected process A for onward studies. FTIR spectra for these SeNPs and the raw materials are presented in Figure S6 and clearly evidence the successful formation of surface-functionalised SeNPs.

### 3.2. Antioxidant Activity

DPPH assays showed that the synthesised TPGS-SeNPs have strong potential (up to 82% relative scavenging value) to scavenge free radicals, with comparable activity to AA (85%) at concentrations around 10 µg/mL (Figure S7). Over the range 0.2–20 µg/mL, there was no significant difference in scavenging activity (*p* = 0.2) between the TPGS-SeNPs and AA itself. This is in accordance with literature findings [63,64].

### 3.3. Cell Viability Results

The toxicity of the TPGS-SeNPs was initially investigated using the PrestoBlue assay and ARPE-19 cells. The nanoparticles were prepared freshly, NP numbers were measured on the day of cell culture using DLS, and then the stock was diluted to prepare stock solutions. Across the concentration range from 1 to 157 μg/mL Se, viability remains above 67% (Figure S8). The IC_50_ value is found to be 524 μg/mL.

**Figure 3:**
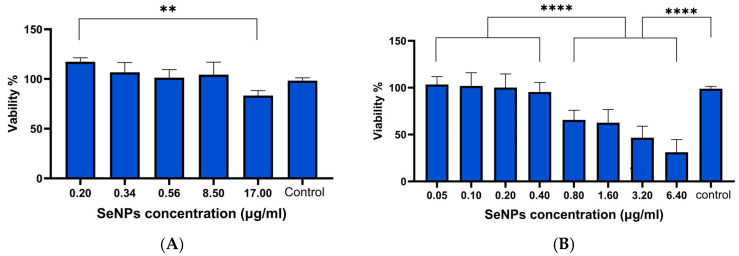
(**A**) Viability of ARPE-19 cells after treatment with SeNPs at various concentrations. The graph shows a biocompatible concentration up to 17 µg/mL with viability of 83%. (**B**) The viability of HLE cells exposed to SeNPs. Doses up to 0.4 μg/mL are well tolerated by the cells, while at concentrations of 0.8 μg/mL and beyond, a significant decrease in viability was observed, with IC_50_ = 2.2 µg/mL. Data are presented as mean ± SD **** *p* < 0.0001, ** *p* < 0.01 (*N* = 3, *n* = 6).

The toxicity of the TPGS-SeNPs was investigated in more detail using both ARPE-19 and HLE cells. Figure 3 reveals that ARPE cells are less sensitive to SeNPs than HLE cells. At concentrations of 8.5 μg/mL or below, ARPE cells showed >95% viability and no significant difference compared to the untreated cells control. In contrast, HLE cells had a biocompatible dose ≤ 0.4 µg/mL. The calculated IC_50_ was 2.2 µg/mL. All concentrations ≤0.4 µg/mL showed significantly greater viability than higher doses (≥0.8 µg/mL; *p* ≤ 0.001). The IC_50_ value of 2.2 μg/mL for HLE cells demonstrates high sensitivity to SeNPs, as relatively low concentrations start to exhibit significant cytotoxic effects.

The PrestoBlue results were confirmed with ATP assays, where a significant reduction in ATP production with increasing SeNP concentrations compared to the control group (Figure S9; *p* < 0.0001) was seen at higher concentrations. At the lowest concentration (0.1 µg/mL), the luminescence remained comparable to the control. However, at concentrations of 0.2 µg/mL and above, a substantial and dose-dependent decrease in ATP production was observed.

**Figure 4:**
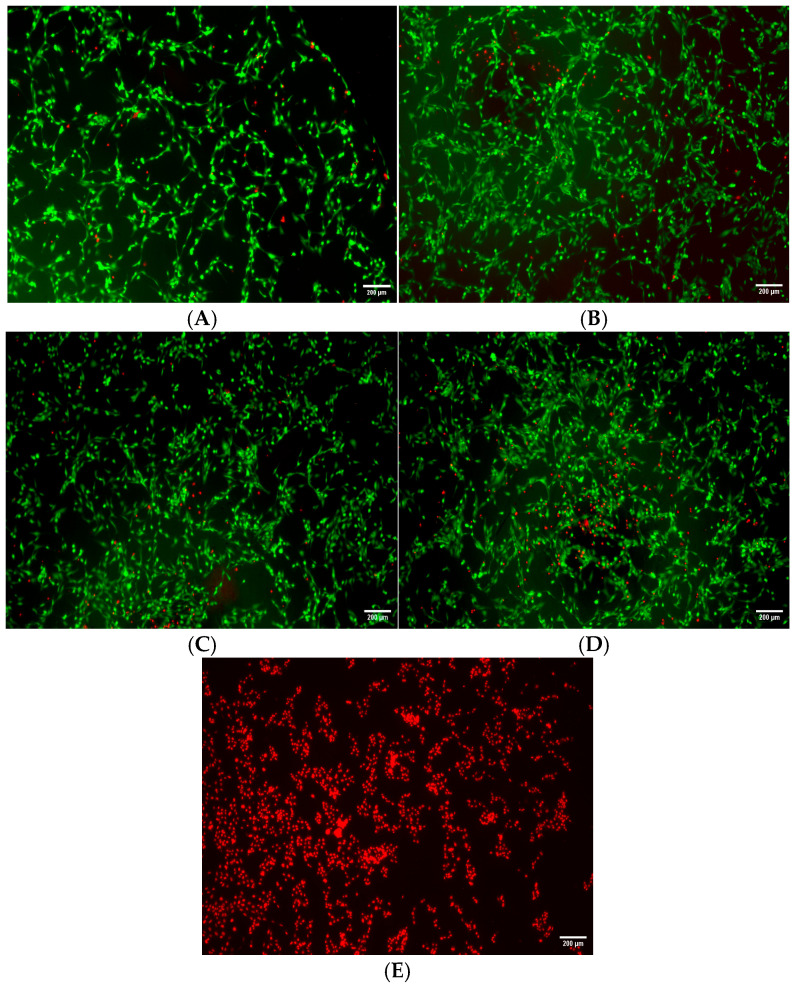
Cell viability of HLE cells as viewed using live–dead imaging following treatment with various SeNP concentrations and 300 µM H_2_O_2_. SeNP concentrations are as follows: (**A**) 0.2 µg/mL; (**B**) 0.1 µg/mL; (**C**) 0.05 µg/mL; (**D**) 0 µg/mL; (**E**) HLE cells incubated with ethanol. Scale bar: 1000 μm.

An investigation was next carried out to determine the SeNPs’ ability to protect against hydrogen peroxide-mediated oxidative stress. In this test, HLE cells were co-incubated with biocompatible concentrations of SeNPs along with hydrogen peroxide for a duration of 24 h. Figure 4 depicts the resultant images, where both live (green) and dead (red) cells can be seen. The cells treated with SeNPs exhibited a slightly lower number of dead cells in comparison with cells given H_2_O_2_ but no selenium, with this effect being dose-dependent and most notable at the highest concentration. This suggests that the nanoparticles have a protective effect against oxidative stress and hydrogen peroxide-induced cellular damage.

### 3.4. GPx Assay Results

**Figure 5:**
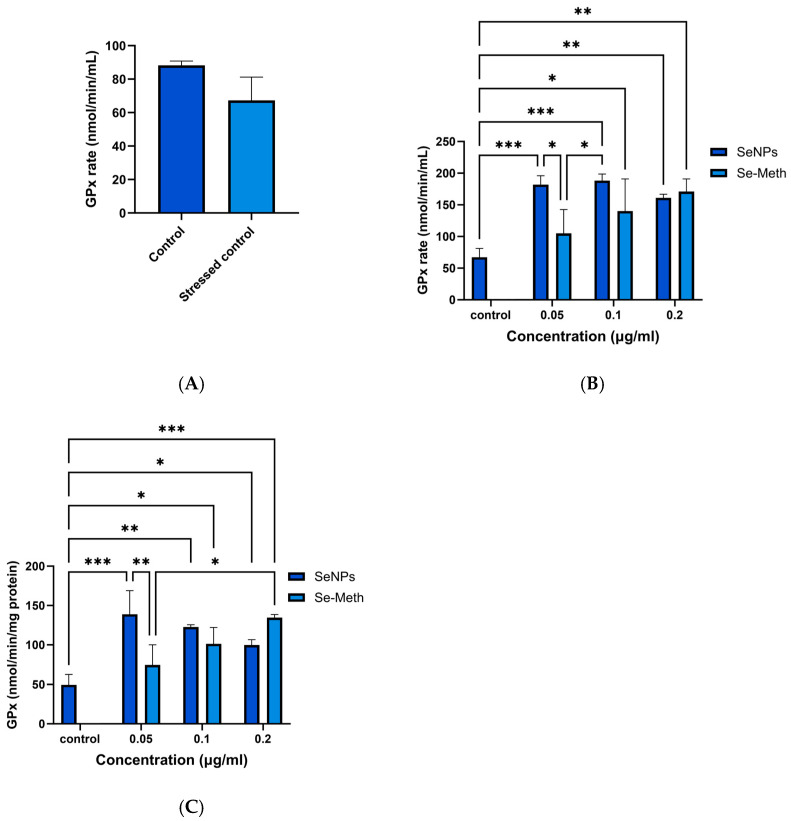
GPx activity results. Activity is expressed as nmol/min/mL. (**A**) GPx activity assay results for control samples. The GPx activity of H_2_O_2_-stressed HLE cells was lower than that of unstressed cells, but there is no significant difference (*p* > 0.05, *N* = 3). (**B**) Raw GPx rate, showing that all SeNP concentrations lead to a significant increase in rate compared to the control, while lower doses of Se-Meth treatment did not show significance. (**C**) GPx activity normalised to the total amount of protein per sample. * *p* < 0.05/** *p* < 0.01/*** *p* < 0.001. Data are presented as mean ± SD (*N* = 3, *n* = 3). In (**B**,**C**) the control comprises HLE cells given H_2_O_2_ but which did not receive any selenium supplementation.

The GPx assay measures GPx activity indirectly, via a coupled reaction with glutathione reductase [65]. A calibration curve was first constructed (see Figure S10). No statistical difference in GPx activity was seen between healthy and stressed cells in the absence of selenium supplementation (Figure 5A).

Increased GPx activity can however be seen in stressed HLE cells pretreated with different forms of selenium (Figure 5B). Increases in GPx activity can be observed with soluble Se-methionine (Se-Meth), but the maximum level of GPx activity is generally lower compared to that attained with equal doses of SeNPs. The maximum activity seen for Se-Meth is at a concentration of 0.2 µg/mL. The GPx activity appears to be inversely correlated with the concentration of SeNPs, and the lowest tested dose (0.05 µg/mL) was sufficient to increase GPx activity (*p* < 0.001 compared to control), with greater activity here than with higher doses. Both SeNPs and Se-Meth thus boost GPx activity, but SeNPs achieve statistically greater concentrations except at the highest dose tested (Figure 5B).

The GPx rate was next normalised to total protein concentration, using concentrations obtained via the BCA method. This was undertaken to take into account any variation in protein concentration across the samples. The data (Figure 5C) thus reflect the proportion of the total protein content which is GPx. Again, there is a significant increase in GPx rate when the particles are treated with SeNPs or Se-Meth, and at low concentration (0.05 μg/mL), the nanoparticles are more effective than Se-Meth (*p* < 0.01). At concentrations of 0.1 μg/mL or above, there is no significant difference between the nanoparticles and Se-Meth at the same dose. All doses showed significance against the control (*p* < 0.05), except Se-Meth at 0.05 μg/mL.

Overall, it is clear that supplementation with Se is associated with elevated GPx activity in vitro in HLE cells. The greatest activity is seen with a low concentration of SeNPs, and increasing the SeNP concentration does not lead to substantial increases in GPx rate.

### 3.5. GSH Assay Results

Glutathione is a tripeptide composed of glutamyl–cysteine–glycine. It has a crucial function in protecting the human lens from damage caused by oxidative stress [66,67]. Preserving GSH levels in the lens could be essential for preventing cataract formation [66]. Thus, we aimed to examine the impact of SeNPs on GSH levels in the HLE cell line after a 24 h exposure to the nanoparticles, using the GSH/GSSG assay. A GSH calibration curve was first constructed (Figure S11).

**Figure 6:**
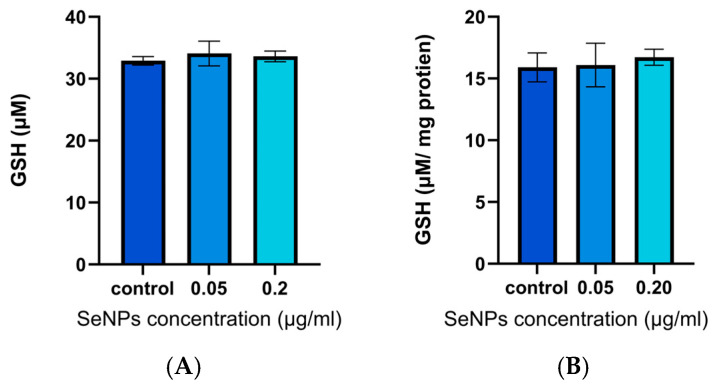
The effects on GSH of exposing HLE cells to SeNPs for 24 h at 0.05 and 0.2 μg/mL and then applying H_2_O_2_ stress. (**A**) Raw GSH concentrations. (**B**) GSH levels normalised to total protein content as determined using the BCA assay. No significant differences are observed in either case (*p* > 0.05, *N* = 4, *n* = 2).

Exposing HLE cells to SeNPs at doses of 0.05 and 0.2 μg/mL for 24 h after applying H_2_O_2_ stress did not provide a significant elevation in GSH levels relative to the negative control group, which received no SeNPs (Figure 6A). When normalising GSH levels to the total protein content using the BCA assay, again there was no significant difference observed (Figure 6B). These findings suggest that SeNP treatment does not contribute to increased GSH activity in HLE cells under stress, although the same dose significantly increased GPx enzyme activity under the same conditions. These unchanged GSH levels can be attributed to various factors, such as a sub-optimal incubation time for GSH activity, and the balance between GSH synthesis and consumption [68].

### 3.6. MDA Assay Results

**Figure 7:**
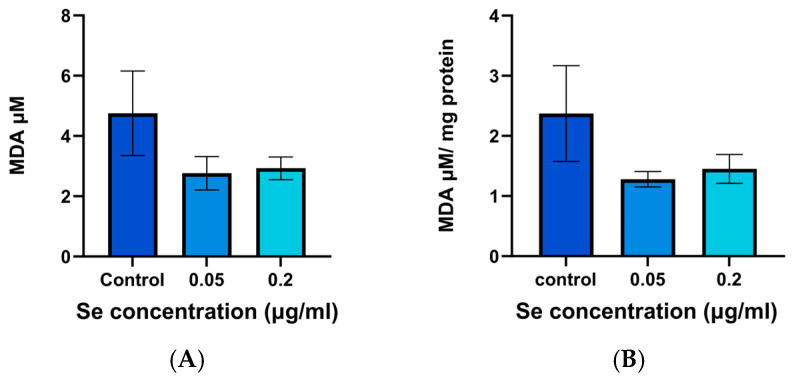
MDA assay data for H_2_O_2_-stressed cells (control), as well as for cells that were pretreated with 0.05 µg/mL and 0.2 µg/mL of SeNPs and then subject to the same stress. (**A**) MDA concentrations (µM). (**B**) MDA levels normalised to the total protein content, determined using BCA assays. Data are presented as mean ± SD (*N* = 3, *n* = 3). No significant differences were found (*p* > 0.05).

This study examined the influence of SeNPs on MDA levels. These serve as an indicator of lipid peroxidation and thus can give an idea of the response of cells exposed to oxidative stress. Figure 7 illustrates the MDA levels observed in SeNP augmentation assays, calculated based on the calibration curve in Figure S11. HLE cells pretreated with both 0.05 and 0.2 µg/mL SeNPs followed by H_2_O_2_ showed apparently lower MDA levels relative to the non-pretreated HLE cells, but this difference was not significant, even after normalising MDA levels to the total protein content (Figure 7A,B). Under these conditions, it thus appears that the SeNPs are not able to exert an effect on MDA production.

### 3.7. TrxR Assay Results

**Figure 8:**
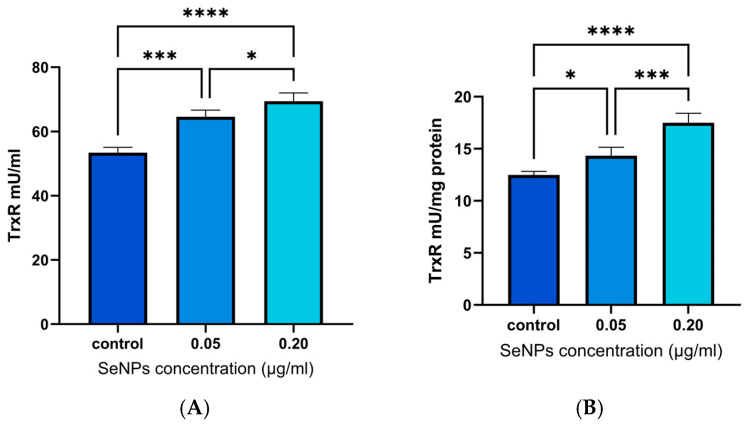
Thioredoxin reductase activity levels in cells exposed to oxidative stress, with and without SeNPs pretreatment. (**A**) TrxR concentrations. (**B**) TrxR levels normalised to total protein content, determined using BCA assays. * *p* < 0.05/*** *p* < 0.001/**** *p* < 0.0001. Data are presented as mean ± SD (*N* = 4, *n* = 2).

This assay assessed the impact of SeNPs on the activity of TrxR in cells subjected to oxidative stress. TrxR is an essential enzyme in the thioredoxin system, responsible for preserving cellular redox equilibrium and diminishing oxidative stress [22]. The results are shown in Figure 8.

The data show a significant increase in TrxR activity in cells pretreated with with 0.05 and 0.2 µg/mL of SeNPs, relative to the control without NP treatment. This illustrates selenium’s efficacy in enhancing cellular antioxidant defenses. This occurs in a dose-dependent manner.

### 3.8. 3-AT Assay Results

**Figure 9:**
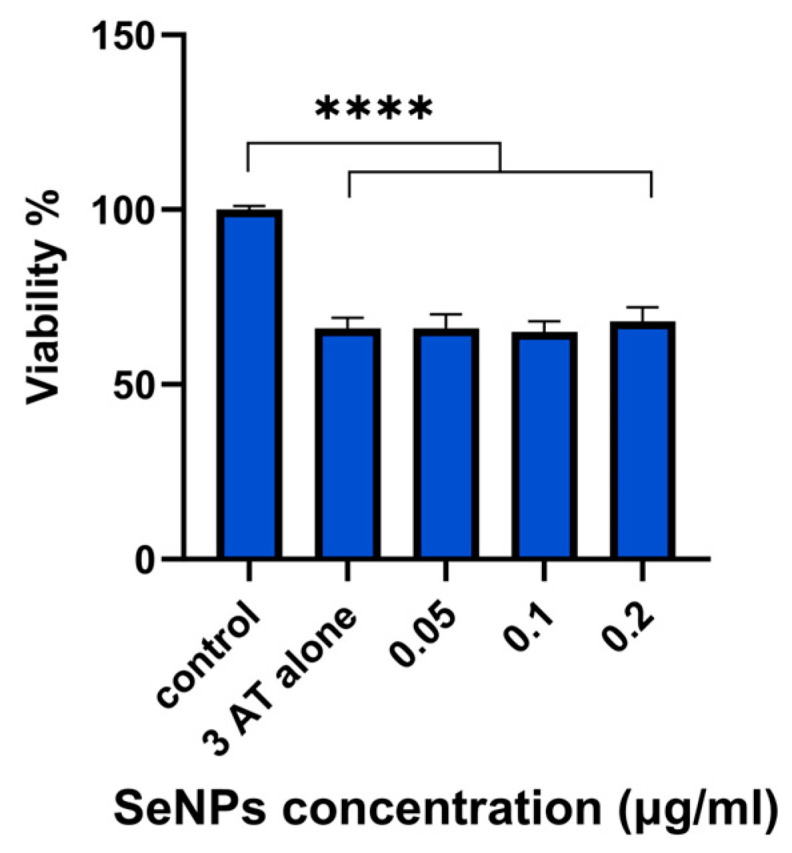
Catalase-mimetic activity of SeNPs in HLE cells (without H_2_O_2_ treatment). Cell viability is measured as a percentage of the control (untreated cells). SeNPs do not show any protection against the damage induced by the catalase inhibitor 3-AT (100 mM), **** *p* < 0.0001. Data are presented as mean ± SD (*N* = 4, *n* = 5).

Catalase is a vital enzyme which helps to protect cells against the effects of H_2_O_2_ [27]. 3-AT was used in this study to suppress catalase function in the HLE cell line, which results in buildup of intracellular H_2_O_2_ and, eventually, cellular death [61]. These experiments allow us to assess the capacity of SeNPs to compensate for the activity of catalase, reprising the ability of the latter to protect against intracellular H_2_O_2_. The assay results are presented in Figure 9.

Cells administered 3-AT alone exhibit a marked decrease in cell viability, highlighting the detrimental impact of catalase inhibition (*p* < 0.0001). The viabilities seen with all SeNP concentrations are the same as those with 3-AT alone. The data thus reveal that SeNPs alone are not able to reprise the activity of catalase when this is inhibited by 3-AT (*p* < 0.0001). SeNPs likely do not directly mimic catalase’s enzymatic function. Instead, their antioxidant role may be mediated through other pathways, such as enhancing GPx and TrxR activity. This emphasises that SeNPs do not act as a general replacement for all enzymatic antioxidants. The inability to restore catalase activity highlights the importance of the endogenous catalase system in neutralising intracellular H_2_O_2_.

## 4. Discussion

The solvent selection clearly impacts the growth of TPGS-SeNPs, ultimately determining their size. Throughout our experiments, we maintained a TPGS concentration at 0.05% *w*/*v* (0.33 mM), exceeding the critical micelle concentration of 0.02% in aqueous media to ensure adequate stabilisation potential [69]. In process A, the introduction of TPGS prior to AA enables effective stabilisation of the selenium precursor during reduction, and subsequently smaller particles are formed [70]. TPGS can interact directly with the Se precursor, forming a stabilised environment for nucleation. The subsequent gradual addition of ascorbic acid regulated the reduction rate, mediating nucleation dynamics to minimise polydispersity [71]. The presence of ethanol in process B appeared to reduce the interaction efficacy between TPGS and sodium selenite [72,73]. This results in less efficient stabilisation during nucleation and a slight increase in particle size. Process C, using a thin-film rehydration methodology, produced substantially larger SeNPs than the other approaches. This likely results from different nucleation dynamics when a sodium selenite solution rehydrates a TPGS thin film before reduction with AA. The spatial arrangement of TPGS molecules in the film structure appears to create nucleation conditions that favour larger particle formation, possibly through altered local concentrations of reactants or modified interfacial energies.

Our results demonstrate that TPGS effectively functions as a surface stabiliser for selenium nanoparticles to limit particle growth compared to uncoated formulations. Despite this initial stabilisation, our stability studies revealed gradual size increases during storage. This suggests that additional stabilising agents may be necessary to maintain particle dimensions below the critical 200 nm threshold thought to be required for effective ocular permeation [74].

DPPH results are broadly consistent with other studies described in the literature [63,64]. However, the EC_50_ (concentration required to inhibit 50% of free radicals) for the TPGS-SeNPs was found to be 1.55 µg/mL, notably lower than previously reported for SeNPs prepared with ginger extract (estimated to be 125 μg/mL) [75]. This enhanced radical scavenging efficiency corroborates the influential role of the TPGS surface coating in affecting antioxidant activity [62,76]. Comparative assessment of bare SeNPs proved challenging due to their inherent colloidal instability, highlighting a common limitation in evaluating uncoated nanoparticle systems [77,78].

A cytotoxicity assessment identified the biocompatible doses of the SeNPs for both HLE and ARPE-19 cell lines, establishing a foundation for further exploration of their antioxidant effects. The former cells were much more susceptible to SeNP-induced death than the latter. It is well established that nanoparticles have varying toxicity towards different cell lines [79]. A study conducted by Zhong et al. [14] concluded that a safe SeNP concentration on HLE cells was 0.04–0.31 μg/mL, showing similar biocompatible concentrations to the results obtained here (0.05–0.4 μg/mL) but using a different coating compound (*Lycium barbarum* polysaccharide) [14]. This small discrepancy is to be expected, given that it is known that the cytotoxicity profile of SeNP formulations in vitro varies depending on the type of coating and cell line [79,80,81]. In other work, Guisbiers et al. investigated the cytotoxicity of SeNPs on ARPE-19 cells. Their findings revealed that 70% of the cells remained viable when exposed to 50 μg/mL of selenium nanoparticles synthesised via pulsed laser ablation in deionised water [82]. Guisbiers’ study showed more toxic effects of SeNPs on APRE cells than the results obtained here, where a concentration of 157 μg/mL led to a viability of 68%. These disparities could be due to differences in particle size and synthetic methods [83].

A comparative study aiming to assess the toxicity of different coatings of SeNPs on several cell lines showed that SeNPs with positively charged coatings exhibited a higher level of toxicity, while a negative surface charge led to notably lower levels of toxicity [80]. Research has shown that the electrical charge on the surface of nanoparticles significantly influences their interaction with cell membranes, which in turn affects cellular uptake and subsequent biological responses [84,85]. Nanoparticles with a positive charge are often linked to increased cellular uptake, because of their electrostatic attraction with the negatively charged cell membrane. Very high levels of internalisation may result in oxidative stress and cytotoxicity [85,86]. In contrast, nanoparticles that are negatively charged show reduced cellular uptake and lower levels of cytotoxicity [87,88]. Hence, modifying the surface charge on SeNPs to be negative (−15 mV) is a feasible approach to regulate their biological functions. That said, in many cases, the particles will need to be internalised to be active, and thus there is a balance to be struck between the rate and extent of uptake and the need to preserve cellular viability. In this study, NP uptake was clearly indicated by the changes in enzyme activities observed post-treatment.

Increasing concentrations of SeNPs (from 0.1 μg/mL to 8 μg/mL) were found to lead to a decrease in ATP production. This indicates the nanoparticles could potentially impair mitochondrial function, particularly at elevated doses [89]. The reduction in ATP levels may be linked to the cytotoxic effects of SeNPs on cellular metabolism, as documented in several studies [63,76,79]. Reduced ATP production is detrimental to cellular viability as ATP is essential for numerous energy-dependent processes, including ion transport, protein synthesis, and maintaining cellular homeostasis. The inhibitory effect of SeNPs on ATP production, particularly at higher concentrations (>0.2 μg/mL), may therefore compromise cell survival. This underscores the delicate balance required in using SeNPs therapeutically, as their dose-dependent effects on mitochondrial function can have profound implications for cellular health.

Based on the above assay results, NP concentrations ˂ 0.4 µg/mL were studied further. The effects of biocompatible doses of SeNPs (0.05–0.2 µg/mL) on oxidative stress were evaluated in a live/dead assay. HLE cells were stressed through exposure to hydrogen peroxide. SeNPs exhibited a protective effect, reducing the number of dead cells and suggesting their potential involvement in augmenting cellular resistance to oxidative damage. This agrees with a number of literature studies [90,91,92,93,94].

SeNPs were next assessed for their antioxidant activity in vitro, which was demonstrated by an increase in GPx enzyme levels under oxidative stress conditions. Lower doses of nanoparticles (0.05 µg/mL) led to a greater increase in GPx activity compared to higher doses (0.2 µg/mL). Consistent with these observed results, a literature study explored GPx activity in rats and found that higher selenium concentrations led to reduced GPx activity [95]. This may be attributed to the induction of GPx activity exhibiting saturation characteristics, as reported in the literature [96], and the fact that higher NP concentrations can be toxic [97]. Importantly, significant uplifts in GPx production are seen at concentrations of NPs which are found not to adversely affect cellular ATP production (see Figure S9). The lack of statistical difference in GPx activity between healthy and stressed cells when selenium supplementation was absent (Figure 5A) could be explained by there being insufficient cellular selenium concentrations present to support the upregulation of GPx expression in response to H_2_O_2_ stress

The source of selenium has an important effect on GPx activity. Both SeNPs and Se-Meth boosted GPx activity in HLE cells in this work, but SeNPs showed significant increases at lower concentrations. Many studies indicate that the biological activity of selenium correlates with its chemical form (Se^2−^), and that a nanoscale form (cf. selenium in solution) helps cells to express higher levels of GPx than the organic form [98,99,100]. This could be due Se-Met being non-specifically integrated into proteins, as it readily substitutes for methionine during protein synthesis, and thus not all is available for GPx production [101,102,103,104]. The relative extent of uptake of SeNPs and Se-Met will also play a role here.

Although SeNPs demonstrated antioxidant effects in terms of enhancing GPx enzyme activity, the tested doses did not lead to an increase in GSH enzyme. These apparently contradictory findings can be attributed to various factors, such as inefficient incubation time for GSH activity, and the balance between GSH synthesis and consumption. GPx generally utilises GSH as a reducing agent to catalyse the reduction of peroxides to water [105,106]. Therefore, the elevated GPx activity resulting from SeNP treatment may exceed the cell’s GSH maintenance capacity, resulting in a steady or falling GSH pool [105].

The SeNPs seemed to lead to a reduction in MDA concentration, but no significant differences were observed. This indicates that under the conditions explored in this work there was no effect on lipid peroxidation. This can be rationalised based on the timeframes for the various biological processes, and based on the fact that the impact of selenium varies depending on the dosage [107]. The nanoparticles used may be sufficient to boost GPx activity but insufficient to comprehensively neutralise the ROS responsible for initiating lipid peroxidation. GPx is indirectly effective in reducing lipid hydroperoxides, but non-enzymatic antioxidants like vitamin E and GSH directly reduce lipid hydroperoxides in the cell membrane [1,108,109]. If the concentration of these antioxidants is insufficient, MDA levels may remain unchanged [108]. MDA is also a stable end-product of lipid peroxidation, and hence it may persist even when SeNPs do reduce ROS production [110,111]. As noted before, the effectiveness of selenium is greatly affected by the amount used and the conditions inside the cells [112].

Selenium is necessary for TrxR to perform its catalytic role effectively [113]. Research indicates that selenium supplementation may upregulate the expression and activity of TrxR in a dose-dependent manner [7,114]. The results reported here agree with previous studies, demonstrating a significant increase in TrxR activity with high-concentration SeNPs. The different responses seen in GPx activity and TrxR expression in the results obtained could be related to cell-specific selenoprotein expression [115]. This is consistent with the idea of a ‘selenium threshold’ for optimum enzyme activity, discussed by Steinbrenner et al. [116].

Despite demonstrable antioxidant activity, SeNPs did not exhibit protective effects against 3-AT-induced inhibition of catalase, which results in H_2_O_2_ build-up and thus oxidative stress. When cells were treated with 3-AT alone, they exhibited a marked decrease in cell viability, highlighting the detrimental impact of catalase inhibition and accumulation of H_2_O_2_. At optimal doses, SeNPs can mitigate oxidative damage caused by H_2_O_2_ accumulation, preserving cell viability [90,91,92,93,94]. This effect is likely mediated by increased GPx and TrxR activity. However, with 3-AT markedly inhibiting catalase activity, the demand for antioxidant defences (like GPx) may surpass the capacity for selenoprotein production, leaving ROS levels inadequately managed.

Reduced GPx and TrxR activity is associated with an increased risk of cataract development [117,118,119]. The ability of the SeNPs to mediate the activity of both suggests their potential as a therapeutic option for managing ocular stress conditions such as cataracts.

## 5. Conclusions

TPGS-SeNPs were prepared using three lab-based processes to give NP sizes ranging from 44 nm to 161 nm. All the NPs were observed to increase in size over time, and all had antioxidant activity comparable to an AA control. Toxicity assessments found a concentration-dependent effect, and that the SeNPs were more toxic to ARPE cells than human lens epithelial cells. In the latter, at concentrations exceeding 0.4 μg/mL, the TPGS-SeNPs caused decreased ATP production and dose-dependent cytotoxicity, suggesting mitochondrial dysfunction. In human lens epithelial cells, TPGS-SeNPs exhibited superior efficacy compared to selenomethionine in enhancing glutathione peroxidase (GPx) activity at lower concentrations, with a saturation threshold identified at 0.05 μg/mL. While the nanoparticles significantly increased thioredoxin reductase (TrxR) activity at higher concentrations, they showed no notable impact on malondialdehyde and glutathione levels at biocompatible doses, indicating a selective influence on enzymatic antioxidant pathways. At concentrations exceeding 0.4 μg/mL, the TPGS-SeNPs caused decreased ATP production and dose-dependent cytotoxicity, suggesting mitochondrial dysfunction. This paper describes the therapeutic promise of TPGS-SeNPs while raising awareness of their concentration-dependent cytotoxicity, advocating for further research to optimise their biomedical applications. The results augment the current literature on the concentration-dependent impacts of SeNPs on cellular metabolism and antioxidant defence systems but also identifies the need for more research in this domain. Developing these nanoparticles into ocular formulations for either topical or injectable applications could thus be a promising direction for future research and clinical applications. To this end, future work should focus on formulation development and the further assessment of these systems both in vitro and in vivo.

## Data Availability

The raw data supporting the conclusions of this article will be made available by the authors on request.

## Supplementary Materials

The following supporting information can be downloaded at https://www.mdpi.com/article/10.3390/pharmaceutics17091157/s1: Additional DLS and TEM data; XRD patterns; IR spectra; DPPH assay results; cell viability of ARPE-19 cells exposed to the SeNPs; ATP assay results; GPx and GSH assay calibration plots.

## Abbreviations

The following abbreviations are used in this manuscript:

ARPE	Adult Retinal Pigment Epithelial
ATP	Adenosine Triphosphate
DLS	Dynamic Light Scattering
DPPH	2,2-Diphenyl-1-picrylhydrazyl
FTIR	Fourier Transform Infrared Spectroscopy
GPx	Glutathione Peroxidase
GSH	Reduced Glutathione
GSSG	Oxidised Glutathione
HLE	Human Lens Epithelial
ICP-OES	Inductively Coupled Plasma Optical Emission Spectrometry
MDA	Malondialdehyde
NADPH	Nicotinamide Adenine Dinucleotide Phosphate
ROS	Reactive Oxygen Species
SeNPs	Selenium Nanoparticles
TEM	Transmission Electron Microscopy
TGA	Thermogravimetric Analysis
TPGS	D-α-Tocopheryl Polyethylene Glycol Succinate
TrxR	Thioredoxin Reductase
XRD	X-ray Diffraction
3-AT	3-amino-1,2,4-triazole
