# Supplementary material for: Selenium Nanoparticles: Synthesis, Stability and In Vitro Evaluation in Human Lens Epithelial Cells
Source: Pharmaceutics. 2025 Sep 3;17(9):1157. doi: 10.3390/pharmaceutics17091157 (PMC12473795; doi:10.3390/pharmaceutics17091157)
Supplement: Supplementary file 1 [file pharmaceutics-17-01157-s001.zip › pharmaceutics-3751357-supplementary.pdf]

Supplementary materials

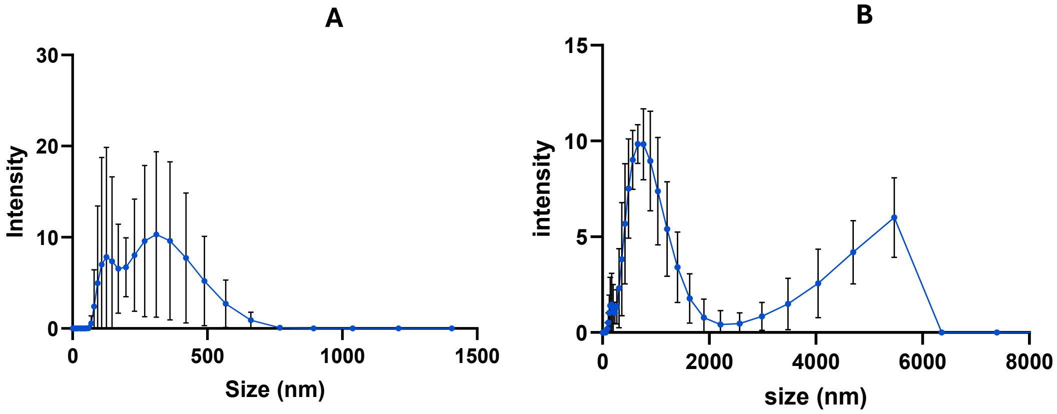

Figure S1: DLS results of uncoated SeNPs showing the size distribution of (A) freshly prepared samples and (B) the same sample after 7 days of storage.

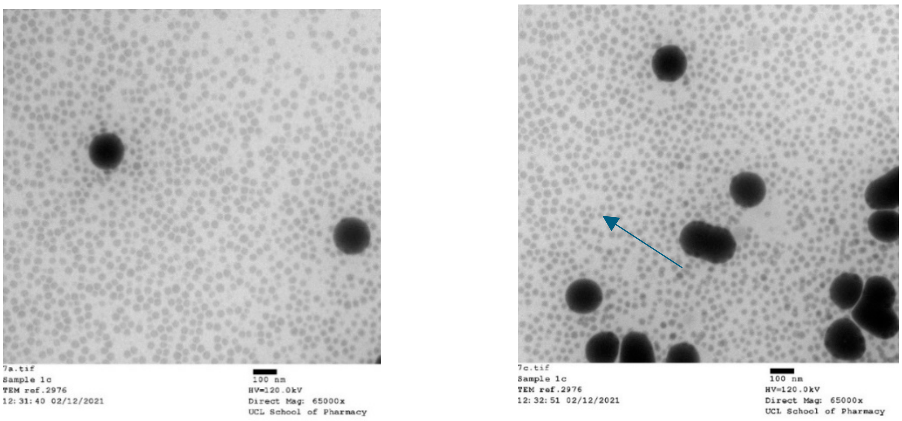

Figure S2. TEM images of the NPs made by process C, showing two different populations of NPs.

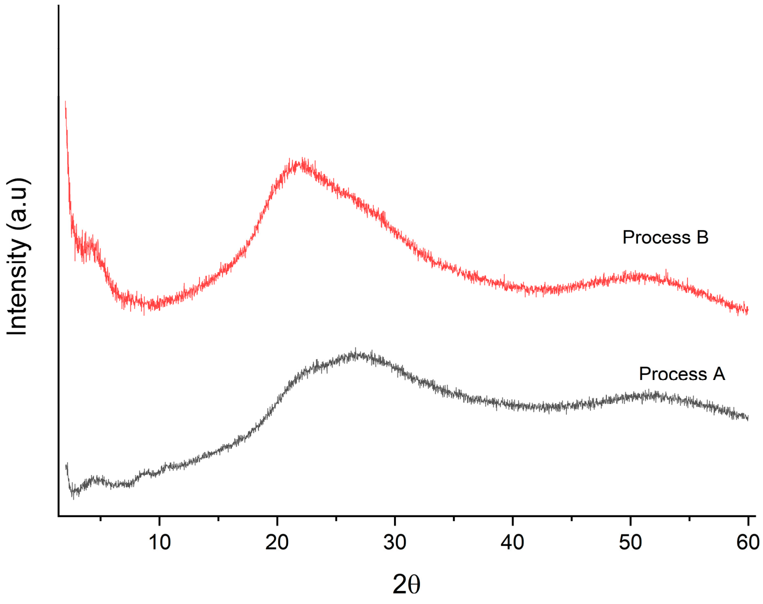

Figure S3. XRD patterns of the process A and B TPGS-SeNPs.

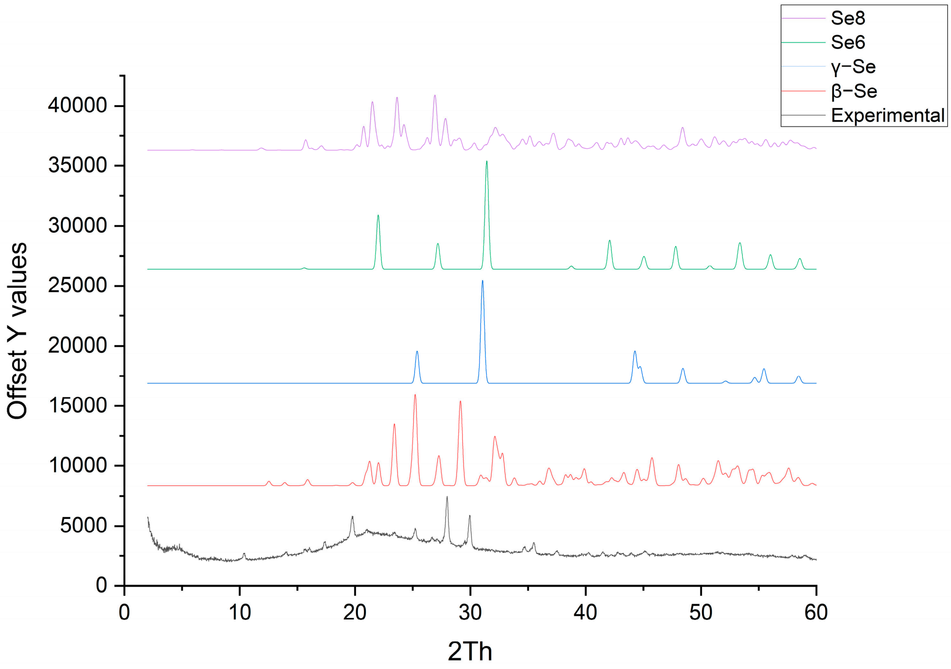

Figure S4. XRD patterns of different Se forms and the process C NPs. Process C was compared to reference patterns of selenium allotropes: Se_8, Se_6, γ-Se, and β-Se. The XRD pattern process C does not match any of the reference crystalline selenium allotropes, indicating distinct structural phase.

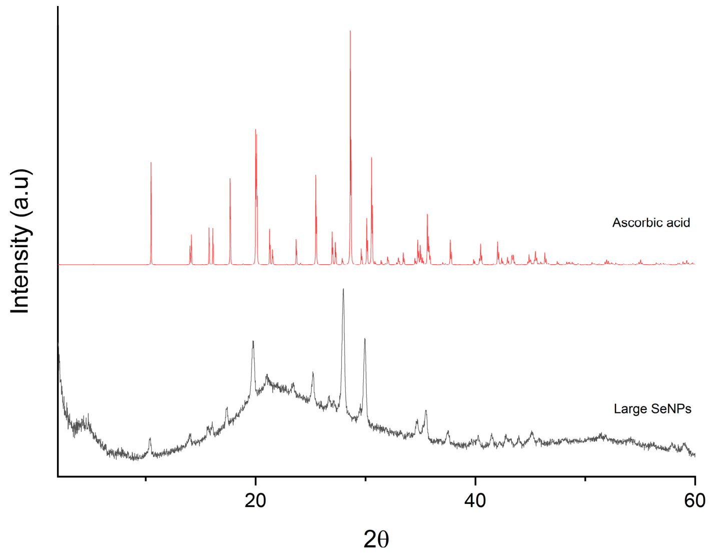

Figure S5. XRD patterns of AA and the process C NPs. The NPs show clear evidence for the presence of crystalline AA in the formulation.

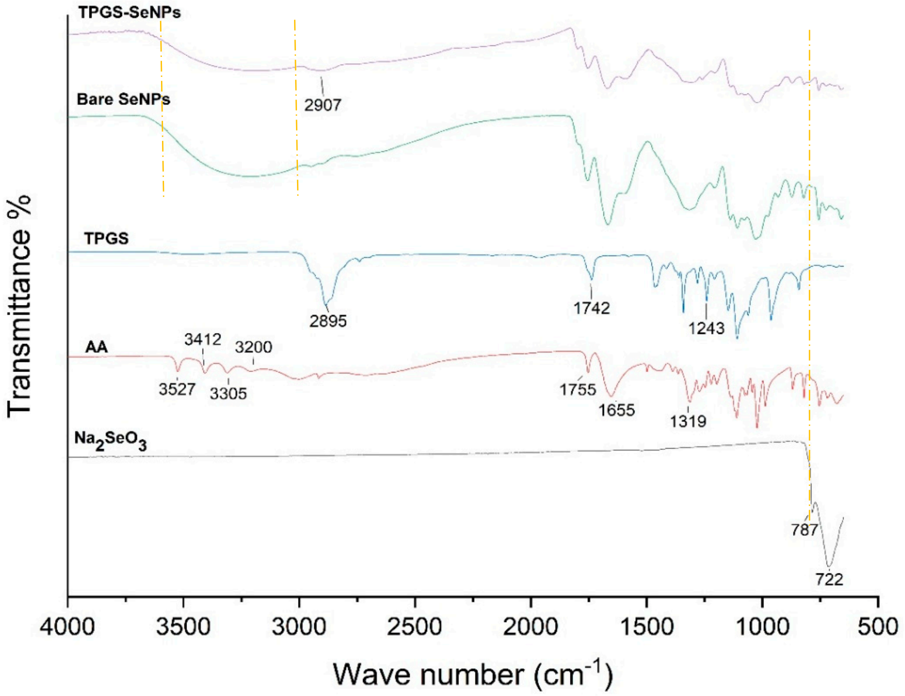

Figure S6. FTIR spectra of the process A TPGS-SeNPs and their components Na_2SeO_3, AA, and TPGS. The characteristic CH_2 stretching band of TPGS was shifted from 2895 cm^{-1} in the raw material to 2907 cm^{-1} in NPs. The C=O stretching vibrations (1755 and 1655 cm^{-1}) from AA are also present in the NPs spectra, with changes in peak shape and position. The sharp, distinct OH stretching peaks from AA (3527, 3412, 3305, and 3200 cm^{-1}) appear significantly broadened and less intense in the nanoparticle formulations. The absence of strong peaks at 787 and 722 cm^{-1} (which are characteristic of Na_2SeO_3) in both nanoparticle formulations is consistent with the reduction of Se(IV) to Se(0).

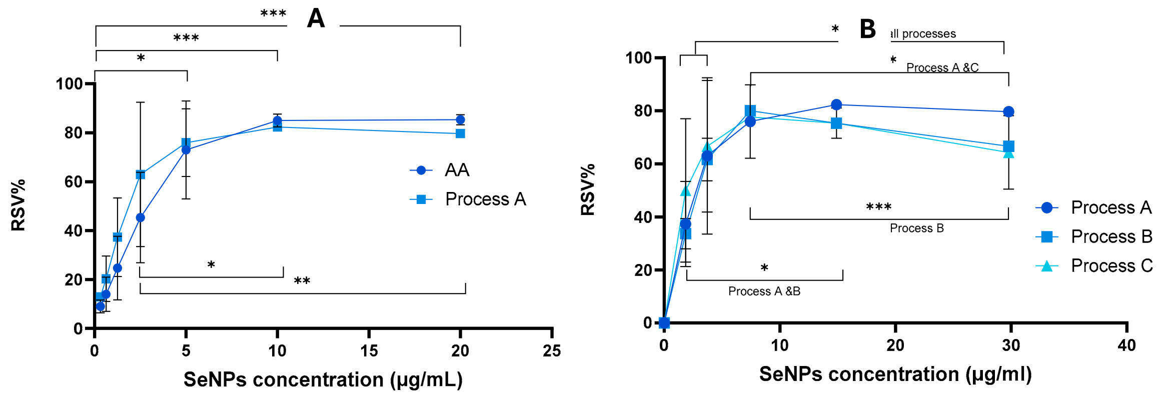

Figure S7. A DPPH assay shows dose dependent antioxidant activity of TPGS-SeNPs obtained by (A) process A (relative scavenging value, RSV%). AA was used as control. EC_{50} calculated: 1.55 μg/mL. (B) DPPH data for NPs from all process (N=3, n=5), two-way ANOVA, Tukey's multiple comparisons. Data are presented as mean ± SD. No Significant differences marked between all processes at the same dose.

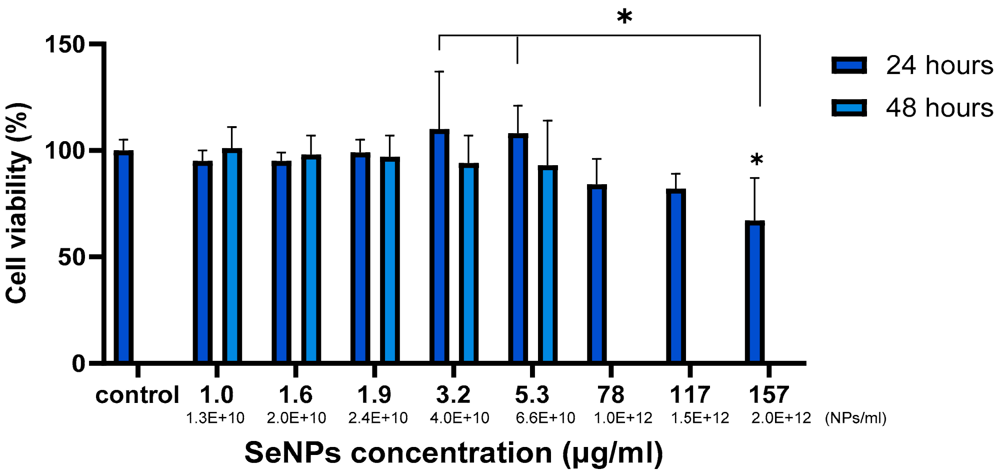

Figure S8. In-vitro cell viability of ARPE-19 cells exposed to different numbers of TPGS-SeNPs prepared by process A. Three independent experiments were performed at different passage numbers, with 5 wells per experiment (N=3, n=5).

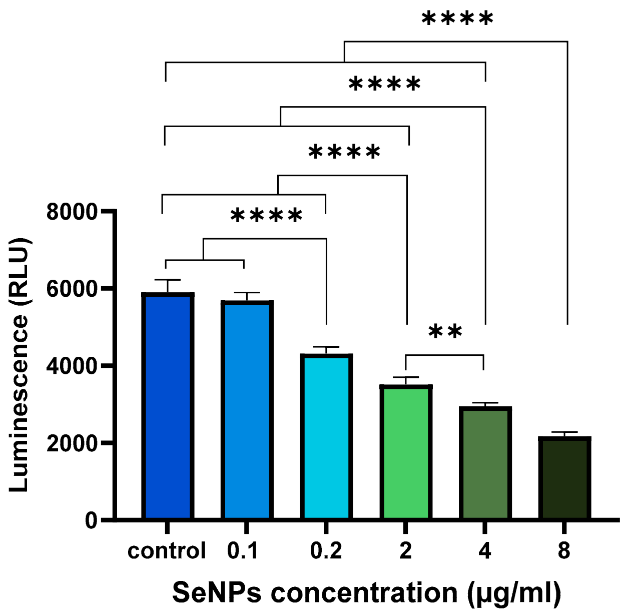

Figure S9. Effect of SeNPs on ATP production in HLE cells. HLE cells were pre-treated with different concentrations of SeNPs for 24 hours. Following the treatment, an ATP assay was conducted to measure ATP production. Data are presented as mean ± SD (N=1, n=4). ****p < 0.0001, **p < 0.01.

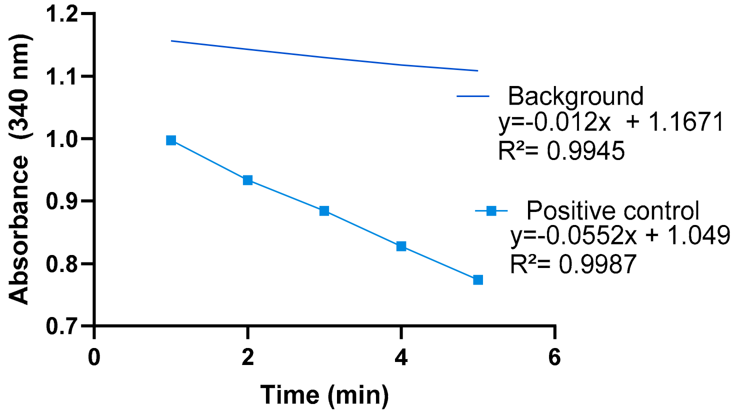

Figure S10. The GPx assay calibration curve. GPx catalyzes the reduction of hydroperoxides using GSH, producing GSSG: R-O-O-H + 2GSH $\xrightarrow{GPx}$ R-O-H + GSSG + H_2O. The GSSG generated is recycled back to GSH by GR, consuming NADPH in the process: GSSG + NADPH + H^+ $\xrightarrow{GR}$ 2GSH + $NADP^+$. NADPH strongly absorbs light at 340 nm, while $NADP^+$ does not. Since NADPH is consumed when GSSG is produced by the GPx reaction, a faster rate of decrease in the absorbance at 340 nm indicates higher GPx activity, as more NADPH is oxidized. The rate of NADPH oxidation (measured as the rate of decrease in absorbance at 340 nm) is proportional to the rate at which GPx is reducing peroxides. The slope of the absorbance decrease thus corresponds to the enzymatic velocity of GPx. The GPx activity may be thus determined using a calibration curve created using a GPx positive control.

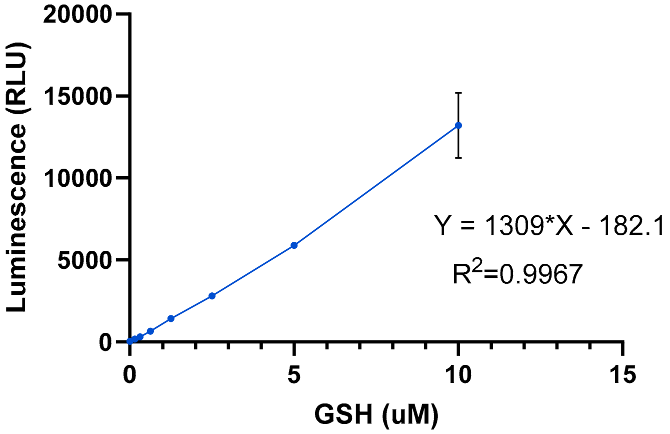

Figure S11. GSH calibration curve (R^2 = 0.996).
